# TRENDS IN REPEAT LACTIC ACID TIMING AND PATIENT OUTCOMES IN A COMMUNITY HOSPITAL IN THE MIDWEST

**DOI:** 10.51894/001c.122892

**Published:** 2024-08-30

**Authors:** Jordan Place, Virginia Labond

**Affiliations:** 1 Emergency Medicine Ascension Genesys

## 35

### INTRODUCTION

One of the most notable outcomes of the Surviving Sepsis Campaign (SSC) is the One Hour Bundle - a collection of specific practice recommendations to be used within one hour of recognizing sepsis. This includes obtaining an initial lactate level and remeasuring this level if the initial level is greater than 2 mmol/L. Currently, there is unclear evidence for the exact timing of the repeat lactic acid (LA) that is most beneficial, with some studies suggesting that earlier testing appears to be superior.

### OBJECTIVES

To investigate relationship between the timing of the repeat LA and outcomes in morbidity and mortality for patients boarding in the ED who meet SIRS criteria and for whom the sepsis bundle was obtained.

### METHODS

This was a single-center retrospective study. Eligible patients were stratified according to the time their LA was repeated (in 2-hour, 4-hour, and 6-hour groups) as well as by their number of comorbidities, with the primary outcome being the final disposition of each patient – whether favorable (discharged home) or unfavorable (subacute rehab, extended care facility, transferred to a tertiary care center, morgue).

### RESULTS

Patients stratified into groups by time (ex. repeat LA obtained within 2 hours vs greater than 2 hours) showed a higher percentage of favorable outcomes for the group with the lower time (ex. 48.1% favorable outcome for LA obtained within 2 hours vs 43.1% for greater than 2 hours). Patients stratified by both time and number of comorbid conditions showed more comparable percentages of favorable outcomes for all groups (ex. for patients with 3 comorbid conditions, 50% favorable outcome for LA obtained within 2 hours vs 48.3% for greater than 2 hours).

### CONCLUSIONS

When stratified only for time, favorable outcomes were consistently higher for groups with lower times to repeat LA. When stratified for time and number of comorbid conditions, there is not a clear trend between the number of comorbid conditions and final disposition.

